# Crystal structure of tetra-μ-acetato-bis­[(5-amino-2-methyl­sulfanyl-1,3,4-thia­diazole-κ*N* ^1^)copper(II)]

**DOI:** 10.1107/S2056989019010272

**Published:** 2019-07-23

**Authors:** Batirbay Torambetov, Shaxnoza Kadirova, Turdibek Toshmurodov, Jamshid Mengnorovich Ashurov, Nusrat Agzamovich Parpiev, Abdukhakim Ziyaev

**Affiliations:** a National University of Uzbekistan named after Mirzo Ulugbek, 100174, Tashkent, Uzbekistan; b Institute of the Chemistry of Plant Substances, Academy of Sciences of Uzbekistan, Mirzo-Ulugbek str. 77, 100170, Uzbekistan; c Institute of Bioorganic Chemistry, Academy of Sciences of Uzbekistan, M. Ulugbek Str, 83, Tashkent, 700125, Uzbekistan

**Keywords:** copper(II), thia­diazole, crystal structure, hydrogen bonding.

## Abstract

The reaction of 2-methyl­thio-5-amino-1,3,4-thia­diazole (Me-SNTD; C_3_H_5_N_3_S_2_) with copper(II) acetate monohydrate [Cu(OAc)_2_·H_2_O; C_4_H_8_CuO_5_] resulted in the formation of the title binuclear compound, [Cu_2_(C_2_H_3_O_2_)_4_(C_3_H_5_N_3_S_2_)_2_] or [Cu_2_(OAc)_4_(Me-SNTD)_2_]. The structure has triclinic (*P* ) symmetry with a crystallographic inversion centre located at the midpoint of the line connecting the Cu atoms in the dimer. These two Cu atoms of the dimer [Cu⋯Cu = 2.6727 (6) Å] are held together by four carboxyl­ate groups. Each Cu atom is further coordinated to the N atom of an Me-SNTD mol­ecule and exhibits a Jahn–Teller-distorted octa­hedral geometry. The dimers are connected into infinite chains by hydrogen bonds between the NH (Me-SNTD) and the carboxyl­ate groups of neighbouring mol­ecules, generating an *R* ^2^_2_(12) ring motif. The mol­ecules are further linked by C—H⋯π inter­actions between the thia­diazole rings and the methyl groups of the acetate units.

## Abstract 2

The reaction of 2-methyl­thio-5-amino-1,3,4-thia­diazole with copper(II) acetate monohydrate resulted in the formation of the title binuclear compound

## Chemical context   

1,3,4-Thia­dazoles are an important class of heterocycles and are of great inter­est because of their broad spectrum of biological activity. 1,3,4-Thia­diazole derivatives and their metal complexes have been shown to display anti­microbial (Önkol *et al.*, 2008 ▸; Abdel-Wahab *et al.*, 2009 ▸; Kadi *et al.*, 2010 ▸), anti­tuberculosis (Karakuşs *et al.*, 2002 ▸; Foroumadi *et al.*, 2004 ▸), anti­oxidant (Chitale *et al.*, 2011 ▸; Sunil *et al.*, 2010 ▸; Khan *et al.*, 2010 ▸), anti­cancer (Padmavathi *et al.*, 2009 ▸; Kumar *et al.*, 2010 ▸;) and anti­fungal (Matysiak *et al.*, 2007 ▸; Klip *et al.*, 2010 ▸; Verma *et al.*, 2011 ▸; Zoumpoulakis *et al.*, 2012 ▸) activities. In addition, some of the 1,3,4-thia­diazole-ring-containing ligands can be efficient uptake agents of toxic metal ions (Mincione *et al.*, 1997 ▸). 1,3,4-Thia­diazo­les also exhibit great potential as pesticides in the fields of herbicides, fungicides, insecticides and even as plant-growth regulators. Their diverse biological activity possibly arises from the presence of the =NCS moiety in the mol­ecule (Oruç *et al.*, 2004 ▸). An inter­esting feature of the metal–ligand chemistry of these compounds is that the complexes can be either mononuclear (Tzeng *et al.*, 2004 ▸; Varna *et al.*, 2018 ▸; Qiu *et al.*, 2014 ▸) or binuclear (Deckert *et al.*, 2016 ▸; Ardan *et al.*, 2017 ▸). A search of the Cambridge Structural Database (CSD Version 5.4, update of February 2019; Groom *et al.*, 2016 ▸) revealed that although crystal structures have been reported for complexes of either 1,3,4-thia­diazole derivatives or OAc with a number of metal ions, including zinc, copper, nickel, manganese, cadmium, cobalt and palladium, no examples are known of mixed-ligand metal complexes containing both 1,3,4-thia­diazole derivatives and OAc. Herein, we report on the synthesis and crystal structure of a new binuclear complex, [Cu_2_(OAc)_4_ *L*_2_], with *L* = 2-methyl­thio-5-amino-1,3,4-thia­diazole (Me-SNTD).
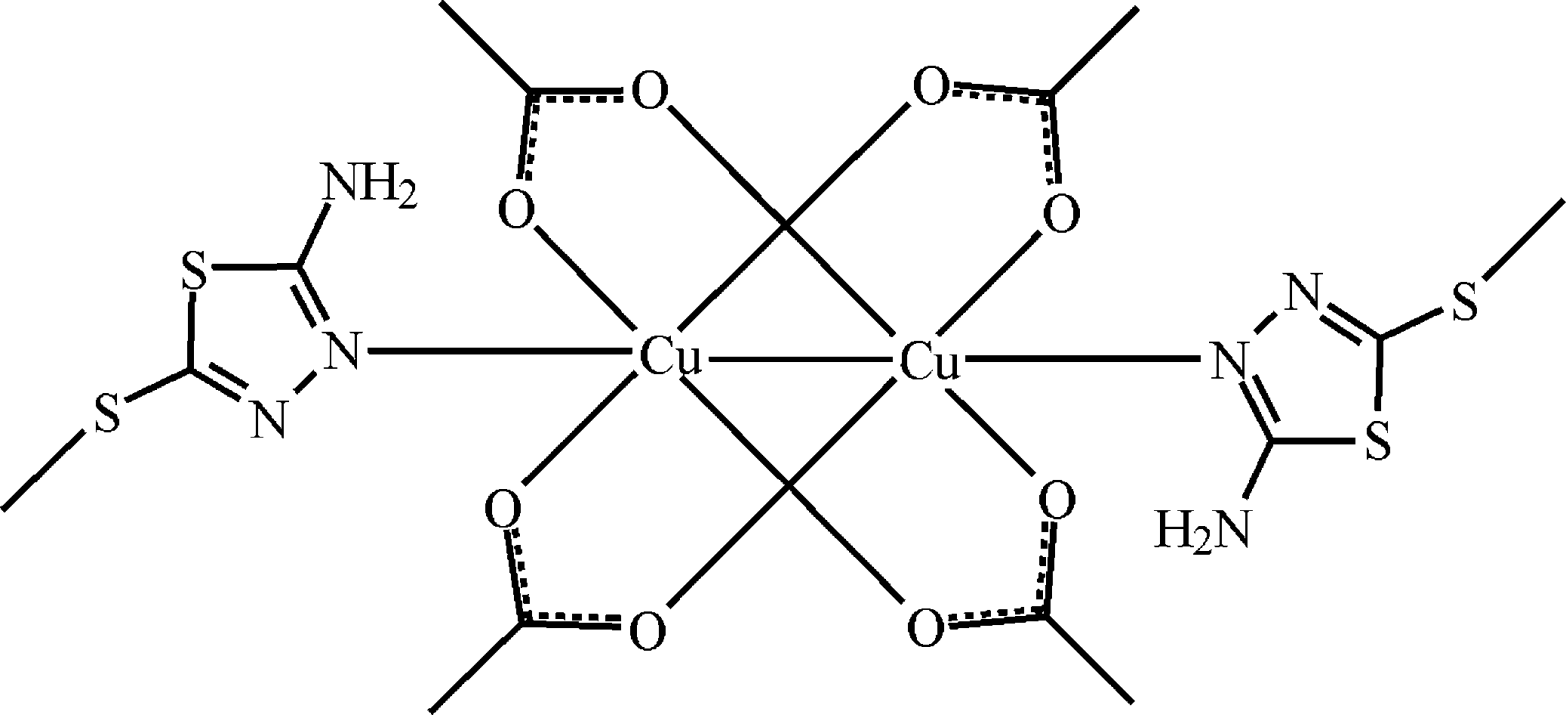


## Structural commentary   

**Figure 1:**
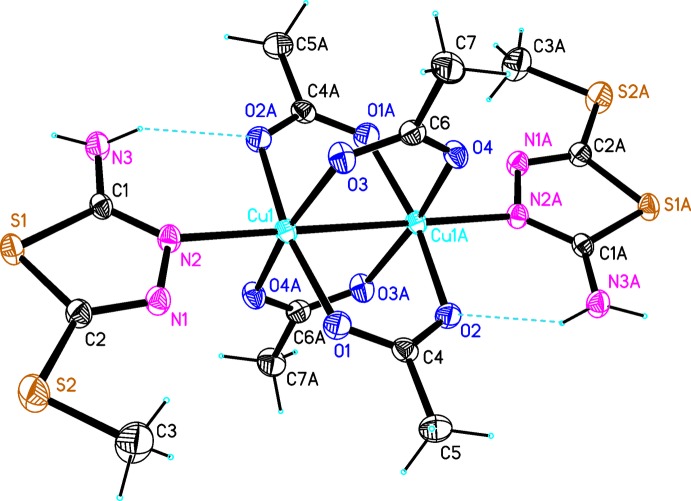
The mol­ecular structure of [Cu_2_(OAc)_4_(Me-SNTD)_2_] with the atom-numbering scheme. Displacement ellipsoids are drawn at the 25% probability level. Intra­molecular hydrogen bonds are shown as dashed lines. Atoms labelled with the suffix A are generated by the symmetry operation 2 − *x*, 1 − *y*, 1 − *z*.

The title binuclear Cu^II^ complex, (I) (Fig. 1 ▸), is arranged about a crystallographic inversion centre located at the midpoint of the Cu⋯Cu-connecting line. The asymmetric unit comprises one half of the complex mol­ecule, namely, one Cu atom, two acetate groups and one 2-methyl­thio-5-amino-1,3,4-thia­diazole mol­ecule. The two Cu atoms in the dimer are held together by the four carboxyl­ate groups. Each Cu atom is bound in a square-pyramidal configuration to four carboxyl­ate O atoms and to the N atom of an Me-SNTD mol­ecule.

Each copper atom is displaced by 0.754 (3) Å from the plane defined by basal-plane atoms O1, O2, O3 and O4 towards the nitro­gen atom, N2. The Cu1*A* —Cu1—N2 angle is 177.95 (7)° [symmetry code: (*A*) 2 − *x*, 1 − *y*, 1 - *z]*. The Cu—O bond lengths range from 1.962 (2) to 2.001 (2) Å and the Cu—N distance is 2.180 (3) Å. The Cu⋯Cu distance is 2.6727 (6) Å and each metal atom exhibits a Jahn–Teller-distorted octa­hedral geometry. The observed Cu—O2 bond length of 1.983 (2) Å is longer than the Cu—O1 distance of 1.962 (2) Å. The elongation of this Cu—O distance may be due to the intra­molecular N3—H⋯O2 hydrogen bond (Table 1 ▸). The conformation of the ligand is approximately planar, with a maximum deviation from the least-squares plane of 0.066 (2) Å for atom N3. The thia­diazole ring is planar (r.m.s. deviation 0.0063 Å). The dihedral angle between the planes of the two independent acetate groups is 82.646 (14)°. The thia­diazole ring is twisted by 18.37 (2)° with respect to the acetate (C4/C5/O1/O2) ligand mean plane.

## Supra­molecular features   

**Figure 2:**
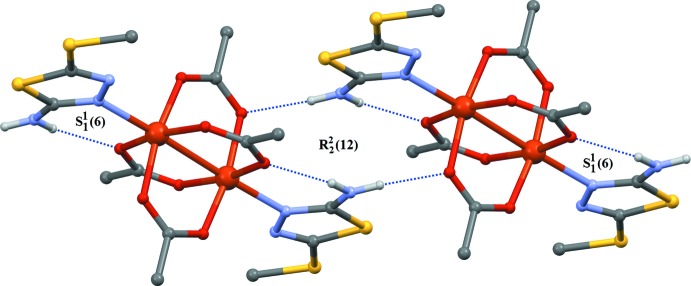
Part of the crystal structure with hydrogen bonds shown as dashed lines. For clarity, H atoms not involved in hydrogen bonding are omitted.

**Figure 3:**
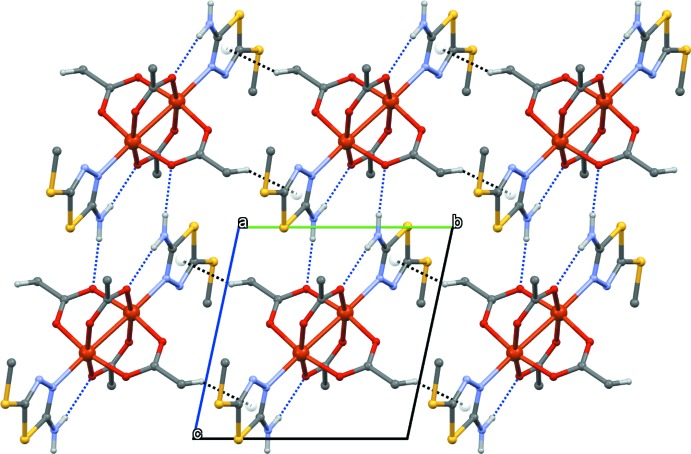
Packing of the structural units in (I). Hydrogen bonds are indicated by blue dashed lines and C—H⋯*π* inter­actions by black dashed lines.

The packing of (I) is shown in Fig. 2 ▸. The acetate group containing oxygen atoms O1 and O3 does not form any hydrogen bonds. However, the acetate group containing oxygen atoms O2 and O4 forms both intra- and inter­molecular hydrogen bonds. Each binuclear complex mol­ecule exhibits one intra­molecular N3—H3⋯O2^i^ hydrogen bond, forming a six-membered ring. The dimers are connected through an inter­molecular N3—H3⋯O4^ii^ hydrogen bond between the NH (Me-SNTD) and the carboxyl­ate groups, forming chains propagating parallel to [001]. The above-mentioned hydrogen bonds give rise to 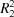
(12), 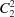
(14) and 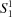
(6) graph-set motifs (Table 1 ▸ and Fig. 2 ▸). Additional C—H⋯*π* inter­actions between the thia­diazole rings and the acetate methyl groups generate a three-dimensional supra­molecular framework (Fig. 3 ▸).

## Database survey   

A survey of the Cambridge Structural Database (CSD Version 5.4, update of February 2019; Groom *et al.*, 2016 ▸) revealed that crystal structures have been reported for complexes of 1,3,4-thia­diazole derivatives and OAc with a number of metal ions, including zinc, copper, nickel, manganese, cadmium, cobalt and palladium. Copper(II) acetate complexes of the general formula [Cu_2_(OAc)_4_ *L*_2_], where *L* is a ligand with an oxygen or nitro­gen ligator atom, have been well explored. The structures of 2-methyl­thio-5-amino-1,3,4-thia­diazole and a complex of this mol­ecule with cadmium have been deposited in the CSD [XUVPEK (Lynch, 2010 ▸) and JIZKEK (Soudani *et al.*, 2014 ▸), respectively]. However, no mixed-ligand metal complexes containing both 1,3,4-thia­diazole derivatives and OAc have been documented in the CSD.

## Synthesis and crystallization   

Cu(OAc)_2_·H_2_O (0.218 g, 1 mmol) and 2-methyl­thio-5-amino-1,3,4-thia­diazole (0.147 g, 1 mmol) were dissolved separately in a mixture of methanol-di­chloro­methane (10 mL, 1:1 *v*/*v*), mixed together and stirred for 1.5 h. The green solid that precipitated was dissolved in methanol to form a green solution. Single crystals of the complex suitable for X-ray analysis were obtained by slow evaporation of the solution over a period of 10 d.

## Refinement   

Crystal data, data collection and structure refinement details are summarized in Table 2 ▸. The restraint N—H = 0.86 ± (1) Å was applied. Methyl H atoms were positioned geometrically C—H = 0.96) and refined as riding with *U*_iso_(H) = 1.5*U*eq(C).

## Supplementary Material

Crystal structure: contains datablock(s) I. DOI: 10.1107/S2056989019010272/cq2032sup1.cif

Structure factors: contains datablock(s) I. DOI: 10.1107/S2056989019010272/cq2032Isup2.hkl

CCDC reference: 1941461

Additional supporting information:  crystallographic information; 3D view; checkCIF report

## Figures and Tables

**Table 1:** Hydrogen-bond geometry (Å, °) *Cg* is the centroid of the S1/N1/N2/C1/C2 ring.

*D*—H⋯*A*	*D*—H	H⋯*A*	*D*⋯*A*	*D*—H⋯*A*
N3—H3*A*⋯O4^i^	0.86 (1)	2.16 (2)	2.963 (4)	156 (4)
N3—H3*B*⋯O2^ii^	0.86 (1)	2.11 (3)	2.884 (4)	150 (4)

Symmetry codes: (i) 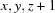; (ii) 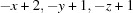.

**Table 2:** Experimental details

Crystal data	
Chemical formula	[Cu_2_(C_2_H_3_O_2_)_4_(C_3_H_5_N_3_S_2_)_2_]
*M*_r_	657.69
Crystal system, space group	Triclinic, *P* 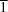
Temperature (K)	571
*a*, *b*, *c* (Å)	8.1069 (4), 8.8955 (4), 9.0421 (5)
α, β, γ (°)	100.656 (4), 98.966 (4), 97.643 (4)
*V* (Å^3^)	624.14 (5)
*Z*	1
Radiation type	Cu *K*α
μ (mm^−1^)	5.70
Crystal size (mm)	0.44 × 0.38 × 0.28

Data collection	
Diffractometer	Rigaku Oxford Diffraction Xcalibur, Ruby
Absorption correction	Multi-scan (*CrysAlis PRO*; Rigaku OD, 2018 ▸)
*T*_min_, *T*_max_	0.083, 1.000
No. of measured, independent and observed [*I* > 2σ(*I*)] reflections	11239, 2582, 2244
*R*_int_	0.052
(sin θ/λ)_max_ (Å^−1^)	0.630

Refinement	
*R*[*F* ^2^ > 2σ(*F* ^2^)], *wR*(*F* ^2^), *S*	0.040, 0.118, 1.07
No. of reflections	2582
No. of parameters	165
No. of restraints	2
H-atom treatment	H atoms treated by a mixture of independent and constrained refinement
Δρ_max_, Δρ_min_ (e Å^−3^)	0.47, −0.44

Computer programs: *CrysAlis PRO* (Rigaku OD, 2018 ▸), *SHELXL* (Sheldrick, 2015 ▸) and *OLEX2* (Dolomanov *et al.*, 2009 ▸).

## supplementary crystallographic information

### Crystal data

**Table:**

[Cu_2_(C_2_H_3_O_2_)_4_(C_3_H_5_N_3_S_2_)_2_]	*Z* = 1
*M**_r_* = 657.69	*F*(000) = 334
Triclinic, *P*1	*D*_x_ = 1.750 Mg m^−^^3^
*a* = 8.1069 (4) Å	Cu *K*α radiation, λ = 1.54184 Å
*b* = 8.8955 (4) Å	Cell parameters from 5762 reflections
*c* = 9.0421 (5) Å	θ = 5.0–75.8°
α = 100.656 (4)°	µ = 5.70 mm^−^^1^
β = 98.966 (4)°	*T* = 571 K
γ = 97.643 (4)°	Block, blue
*V* = 624.14 (5) Å^3^	0.44 × 0.38 × 0.28 mm

### Data collection

**Table:**

Rigaku Oxford Diffraction Xcalibur, Ruby diffractometer	2582 independent reflections
Radiation source: fine-focus sealed X-ray tube, Enhance (Cu) X-ray Source	2244 reflections with *I* > 2σ(*I*)
Graphite monochromator	*R*_int_ = 0.052
Detector resolution: 10.2576 pixels mm^-1^	θ_max_ = 76.2°, θ_min_ = 5.1°
ω scans	*h* = −10→10
Absorption correction: multi-scan (CrysAlisPro; Rigaku OD, 2018)	*k* = −11→11
*T*_min_ = 0.083, *T*_max_ = 1.000	*l* = −10→11
11239 measured reflections	

### Refinement

**Table:**

Refinement on *F*^2^	2 restraints
Least-squares matrix: full	Hydrogen site location: mixed
*R*[*F*^2^ > 2σ(*F*^2^)] = 0.040	H atoms treated by a mixture of independent and constrained refinement
*wR*(*F*^2^) = 0.118	*w* = 1/[σ^2^(*F*_o_^2^) + (0.0726*P*)^2^ + 0.1915*P*] where *P* = (*F*_o_^2^ + 2*F*_c_^2^)/3
*S* = 1.07	(Δ/σ)_max_ = 0.001
2582 reflections	Δρ_max_ = 0.47 e Å^−^^3^
165 parameters	Δρ_min_ = −0.44 e Å^−^^3^

### Special details

**Table:**

Geometry. All esds (except the esd in the dihedral angle between two l.s. planes) are estimated using the full covariance matrix. The cell esds are taken into account individually in the estimation of esds in distances, angles and torsion angles; correlations between esds in cell parameters are only used when they are defined by crystal symmetry. An approximate (isotropic) treatment of cell esds is used for estimating esds involving l.s. planes.

### Fractional atomic coordinates and isotropic or equivalent isotropic displacement parameters (Å^2^)

**Table:**

	*x*	*y*	*z*	*U*_iso_*/*U*_eq_	
Cu1	0.94133 (5)	0.41682 (4)	0.59528 (5)	0.03080 (16)	
S1	0.77895 (10)	0.21114 (9)	1.00281 (9)	0.0416 (2)	
S2	0.42143 (12)	0.08505 (14)	0.83586 (12)	0.0659 (3)	
O4	1.0956 (3)	0.3780 (2)	0.2926 (2)	0.0403 (5)	
O2	0.8200 (3)	0.5370 (3)	0.2953 (3)	0.0409 (5)	
O1	0.7249 (3)	0.3994 (3)	0.4563 (3)	0.0458 (5)	
O3	1.0032 (3)	0.2430 (2)	0.4575 (3)	0.0438 (5)	
N3	1.0840 (4)	0.3406 (3)	0.9585 (3)	0.0442 (6)	
N2	0.8489 (3)	0.2883 (3)	0.7575 (3)	0.0354 (5)	
N1	0.6790 (3)	0.2193 (3)	0.7215 (3)	0.0385 (6)	
C1	0.9196 (4)	0.2903 (3)	0.8988 (3)	0.0327 (6)	
C6	1.0679 (4)	0.2556 (3)	0.3428 (3)	0.0363 (6)	
C4	0.7032 (4)	0.4586 (3)	0.3410 (4)	0.0367 (6)	
C2	0.6271 (4)	0.1756 (3)	0.8362 (4)	0.0383 (6)	
C7	1.1194 (5)	0.1119 (4)	0.2574 (5)	0.0567 (9)	
H7A	1.106850	0.114281	0.150585	0.085*	
H7B	1.048657	0.021473	0.270116	0.085*	
H7C	1.235495	0.108716	0.297339	0.085*	
C5	0.5263 (4)	0.4374 (5)	0.2518 (5)	0.0565 (9)	
H5A	0.459075	0.495634	0.311649	0.085*	
H5B	0.477358	0.329410	0.228270	0.085*	
H5C	0.529261	0.473477	0.158389	0.085*	
C3	0.3241 (6)	0.0719 (6)	0.6412 (5)	0.0740 (13)	
H3C	0.373741	0.001172	0.574724	0.111*	
H3D	0.341504	0.172515	0.616365	0.111*	
H3E	0.204871	0.034989	0.627943	0.111*	
H3A	1.119 (5)	0.357 (4)	1.0554 (14)	0.048 (10)*	
H3B	1.149 (5)	0.377 (5)	0.903 (5)	0.070 (13)*	

### Atomic displacement parameters (Å^2^)

**Table:**

	*U*^11^	*U*^22^	*U*^33^	*U*^12^	*U*^13^	*U*^23^
Cu1	0.0315 (2)	0.0301 (2)	0.0309 (3)	0.00161 (15)	0.00874 (17)	0.00645 (16)
S1	0.0421 (4)	0.0494 (4)	0.0336 (4)	−0.0010 (3)	0.0112 (3)	0.0114 (3)
S2	0.0408 (5)	0.0941 (7)	0.0623 (6)	−0.0141 (5)	0.0082 (4)	0.0347 (6)
O4	0.0516 (13)	0.0353 (10)	0.0367 (12)	0.0105 (9)	0.0164 (10)	0.0054 (9)
O2	0.0312 (10)	0.0497 (11)	0.0409 (12)	0.0015 (9)	0.0043 (9)	0.0130 (10)
O1	0.0333 (11)	0.0572 (13)	0.0450 (13)	−0.0018 (9)	0.0039 (9)	0.0154 (11)
O3	0.0599 (14)	0.0309 (9)	0.0423 (13)	0.0072 (9)	0.0171 (11)	0.0056 (9)
N3	0.0374 (14)	0.0580 (16)	0.0353 (16)	−0.0021 (11)	0.0051 (12)	0.0132 (13)
N2	0.0371 (13)	0.0357 (11)	0.0334 (13)	−0.0002 (9)	0.0077 (10)	0.0106 (10)
N1	0.0375 (13)	0.0409 (12)	0.0373 (14)	−0.0007 (10)	0.0072 (11)	0.0138 (10)
C1	0.0377 (14)	0.0286 (11)	0.0339 (15)	0.0037 (10)	0.0127 (12)	0.0081 (11)
C6	0.0389 (15)	0.0318 (13)	0.0352 (16)	0.0085 (11)	0.0027 (12)	0.0006 (11)
C4	0.0294 (13)	0.0399 (14)	0.0369 (17)	0.0024 (11)	0.0060 (12)	0.0004 (12)
C2	0.0365 (15)	0.0382 (14)	0.0408 (17)	0.0001 (11)	0.0084 (13)	0.0130 (12)
C7	0.077 (3)	0.0424 (17)	0.056 (2)	0.0278 (17)	0.0212 (19)	0.0030 (15)
C5	0.0329 (16)	0.075 (2)	0.059 (2)	0.0039 (15)	0.0028 (16)	0.0144 (19)
C3	0.053 (2)	0.093 (3)	0.067 (3)	−0.015 (2)	−0.004 (2)	0.024 (2)

### Geometric parameters (Å, º)

**Table:**

Cu1—Cu1^i^	2.6728 (8)	N3—H3B	0.856 (10)
Cu1—O4^i^	2.0007 (19)	N2—N1	1.394 (3)
Cu1—O2^i^	1.983 (2)	N2—C1	1.313 (4)
Cu1—O1	1.962 (2)	N1—C2	1.282 (4)
Cu1—O3	1.970 (2)	C6—C7	1.510 (4)
Cu1—N2	2.180 (2)	C4—C5	1.502 (4)
S1—C1	1.745 (3)	C7—H7A	0.9600
S1—C2	1.740 (3)	C7—H7B	0.9600
S2—C2	1.752 (3)	C7—H7C	0.9600
S2—C3	1.789 (5)	C5—H5A	0.9600
O4—C6	1.262 (4)	C5—H5B	0.9600
O2—C4	1.267 (4)	C5—H5C	0.9600
O1—C4	1.251 (4)	C3—H3C	0.9600
O3—C6	1.249 (4)	C3—H3D	0.9600
N3—C1	1.339 (4)	C3—H3E	0.9600
N3—H3A	0.857 (10)		
			
O4^i^—Cu1—Cu1^i^	83.88 (6)	N2—C1—S1	113.1 (2)
O4^i^—Cu1—N2	94.39 (9)	N2—C1—N3	124.8 (3)
O2^i^—Cu1—Cu1^i^	84.10 (7)	O4—C6—C7	117.0 (3)
O2^i^—Cu1—O4^i^	89.30 (9)	O3—C6—O4	125.8 (3)
O2^i^—Cu1—N2	94.80 (9)	O3—C6—C7	117.2 (3)
O1—Cu1—Cu1^i^	82.97 (7)	O2—C4—C5	117.6 (3)
O1—Cu1—O4^i^	89.12 (10)	O1—C4—O2	124.5 (3)
O1—Cu1—O2^i^	167.07 (9)	O1—C4—C5	117.8 (3)
O1—Cu1—O3	90.92 (10)	S1—C2—S2	119.34 (18)
O1—Cu1—N2	98.11 (10)	N1—C2—S1	115.2 (2)
O3—Cu1—Cu1^i^	83.44 (7)	N1—C2—S2	125.5 (3)
O3—Cu1—O4^i^	167.22 (9)	C6—C7—H7A	109.5
O3—Cu1—O2^i^	87.81 (10)	C6—C7—H7B	109.5
O3—Cu1—N2	98.26 (9)	C6—C7—H7C	109.5
N2—Cu1—Cu1^i^	177.95 (7)	H7A—C7—H7B	109.5
C2—S1—C1	86.58 (14)	H7A—C7—H7C	109.5
C2—S2—C3	100.75 (18)	H7B—C7—H7C	109.5
C6—O4—Cu1^i^	122.12 (19)	C4—C5—H5A	109.5
C4—O2—Cu1^i^	122.8 (2)	C4—C5—H5B	109.5
C4—O1—Cu1	125.6 (2)	C4—C5—H5C	109.5
C6—O3—Cu1	124.56 (19)	H5A—C5—H5B	109.5
C1—N3—H3A	121 (3)	H5A—C5—H5C	109.5
C1—N3—H3B	119 (3)	H5B—C5—H5C	109.5
H3A—N3—H3B	119 (4)	S2—C3—H3C	109.5
N1—N2—Cu1	116.61 (18)	S2—C3—H3D	109.5
C1—N2—Cu1	128.89 (19)	S2—C3—H3E	109.5
C1—N2—N1	113.1 (2)	H3C—C3—H3D	109.5
C2—N1—N2	112.1 (3)	H3C—C3—H3E	109.5
N3—C1—S1	122.1 (2)	H3D—C3—H3E	109.5

Symmetry code: (i) −*x*+2, −*y*+1, −*z*+1.

### Hydrogen-bond geometry (Å, º)

*Cg* is the centroid of the S1/N1/N2/C1/C2 ring.

**Table:**

*D*—H···*A*	*D*—H	H···*A*	*D*···*A*	*D*—H···*A*
N3—H3*A*···O4^ii^	0.86 (1)	2.16 (2)	2.963 (4)	156 (4)
N3—H3*B*···O2^i^	0.86 (1)	2.11 (3)	2.884 (4)	150 (4)
C7—H7*B*···*Cg*^iii^	0.96	3.00	3.346 (4)	103

Symmetry codes: (i) −*x*+2, −*y*+1, −*z*+1; (ii) *x*, *y*, *z*+1; (iii) −*x*+2, −*y*, −*z*+1.
